# Supplementary material for: Parasitoid Wasp Acerophagus papayae: A Promising Solution for the Control of Papaya Mealybug Paracoccus marginatus in Cassava Fields in Vietnam
Source: Insects. 2023 Jun 6;14(6):528. doi: 10.3390/insects14060528 (PMC10299258; doi:10.3390/insects14060528)
Supplement: Supplementary file 1 [file insects-14-00528-s001.zip › Table S1 and S2.pdf]

Table S1. Development time of each stage and life cycle of *Acerophagus papaya*

Development stage	Variable (days)	Development stage (days)
		AVG ± SE
Egg	3 - 4	3.7 ± 0.16
1st instar	1 - 2	1.7 ± 0.16
2nd instar	1 - 3	2.0 ± 0.19
3rd instar	1 - 3	2.2 ± 0.22
Total larvae	3 - 5	4.2 ± 0.22
Pre-pupae	2 - 3	2.7 ± 0.16
Pupae	7 - 9	8.0 ± 0.28
Total pupae	9 - 11	10.0 ± 0.28
Total life cycle	15 - 17	16.0 ± 0.32

Note: Host was the P. marginatus at second instar; AVG: average. SE: Standard error; n = 10.

Table S2. Egg-laying of the *Acerophagus papaya*

Day obersvation	Number of egg
1	67
2	61
3	59
4	60
5	57
6	60
7	64
8	63
9	54
10	63
Variable	54 - 67
AVG ± SD	60.8 ± 1.17

Note: AVG: average. SE: standard error. n = 10
